# Web of Science-Based Scientometric Assessment of the Importance of Filtered Water in Dentistry: Spatiotemporal Dynamics, Emerging Patterns, and Collaboration

**DOI:** 10.1155/2024/3279588

**Published:** 2024-05-02

**Authors:** Franco Mauricio, Cesar Mauricio-Vilchez, Diego Galarza-Valencia, Daniel Alvitez-Temoche, Fran Espinoza-Carhuancho, Frank Mayta-Tovalino

**Affiliations:** ^1^Academic Department, Faculty of Dentistry, Universidad Nacional Federico Villarreal, Lima, Peru; ^2^Grupo de Bibliometría, Evaluación de evidencia y Revisiones Sistemáticas (BEERS), Human Medicine Career, Universidad Científica del Sur, Lima, Peru; ^3^Vicerrectorado de Investigación, Universidad San Ignacio de Loyola, Lima, Peru

## Abstract

**Objective:**

The aim of this study was to examine the characteristics of scientific production related to the use of filtered water in the field of dentistry. *Material and Methods*. A quantitative and descriptive observational study was carried out with a scientometric approach. Data were collected from the Web of Science (WOS) database during the period January 1991 to December 2023. A search strategy incorporating a combination of MeSH terms, including terms and thesauri related to “filtered water” and “dentistry”, was used. R Studio version 4.3.2 and CiteSpace 6.2.R7 were used for data analysis.

**Results:**

Over the 32-year study period, 227 scholarly papers from 134 different sources were reviewed. The literature in this field has shown an annual growth rate of 10.44%. During the year 2010, a steady movement in the number of publications and authors was observed, with considerable collaborative interaction. In the year 2020, a large interaction between publications and their citations was found. The “Citation Burst” graph identified three references that have experienced the largest “burst” of citations in an evaluated period. Lotka's law described the productivity of authors, finding that most authors have published only one paper, while a smaller number of authors have published two papers. Most authors contributed a small number of articles, while a few authors contributed a large amount of the existing literature.

**Conclusion:**

A comprehensive overview of the scientific production related to the use of filtered water in dentistry over a span of 32 years is provided. The results highlight the growing interdisciplinarity and international collaboration in this field. Finally, the importance of filtered water in dentistry and its growing relevance in the scientific literature are emphasized.

## 1. Introduction

In dental practices, the use of purified water is crucial for ensuring cleanliness and safety during treatments. Ultrafiltration membranes have emerged as an effective alternative to traditional water purification methods due to their ability to filter out particles and microorganisms [1]. Water, being an essential resource in dentistry, is utilized in a myriad of procedures and equipment for purposes such as cooling and sterilization. However, the potential presence of impurities or contaminants in public water supplies could lead to postprocedural infections. As such, it is imperative that the water employed in dental clinics is of the highest quality [2].

Following dental extraction or any oral surgical procedure, cavities may collect food particles and other debris, potentially disrupting the healing process [3]. Factors such as smoking, inadequate oral hygiene, surgical trauma, and extraction of teeth with preexisting infections or diseases can impact this process. Meanwhile, the quality of tap water available to consumers is determined not only by the treatment it undergoes but also by its journey through the water distribution system [4]. The development of biofilms on pipe walls or on particulate deposits, and their subsequent detachment, can contribute to the degradation of water quality [5, 6].

The occurrence of certain processes is influenced by various factors, including the presence of bacteria, organic matter and nutrients, and residual disinfectants. As such, it is vital to maintain rigorous control over these elements to guarantee water quality [7]. The goal of water filtration in dentistry extends beyond merely eliminating visible impurities; it also aims to remove pathogenic microorganisms that could be harmful to health. This proactive approach to managing the water used in dental practices aids in reducing the presence of bacteria, viruses, and other infectious agents, thereby minimizing the risk of hospital-acquired infections [8].

In the current context, the adoption of suitable filtration systems has become a standard practice in modern dental offices and clinics. These systems not only adhere to regulations but also offer an efficient and trustworthy solution for ensuring the purity of the water utilized in dental procedures [9, 10]. There has been an increased awareness about the significance of water quality in dentistry, leading to the widespread implementation of practices that enhance safety and health in the dental setting [11, 12]. Many dental clinics have instituted a water treatment cycle to guarantee water decontamination.

Scientific production based on the use of filtered water in dentistry is an area of growing interest due to its direct impact on the quality of patient care and safety in dental practices. The choice to investigate this topic is based on the need to better understand current trends, emerging practices, and collaboration patterns in this field. This scientometric study on filtered water in dentistry has significant clinical relevance. By identifying emerging trends and patterns, it can help dental professionals better understand the importance of filtered water in their practices. In addition, it can guide future research and policy in this field to improve patient care [1–9].

Thus, the aim of this study was to examine the characteristics of scientific production related to the use of filtered water in the field of dentistry.

## 2. Materials and Methods

### 2.1. Study Design

A descriptive study with a scientometric approach was carried out to evaluate the characteristics of the scientific production of filtered water in dentistry.

### 2.2. Data Collection

Data were extracted from the Web of Science (WOS) database for the period January 1991 to December 2023. The search strategy was performed on January 15, 2024, and 227 studies potentially related to the topic were found; this strategy included a combination of various MeSH terms, including terms and thesauri related to “filtered water” and “dentistry”. The strategy that was developed was as follows: TS = (“Filtered Water” OR “Purified Water” OR “Clean Water” OR “Purified Drinking Water” OR “Distilled Water” OR “Treated Water” OR “Clarified Water” OR “Processed Water” OR “Refined Water” OR “Crystal-Clear Water” OR “Decontaminated Water” OR “Purged Water” OR “Microfiltered Water” OR “Ultrafiltered Water” OR “Reverse Osmosis Water” OR “Spring Water” OR “Pristine Water” OR “Untainted Water” OR “Safe Drinking Water” OR “Potable Water” OR “Crisp Water”) AND TS = (“Dentistry” OR “Oral and Maxillofacial Surgery” OR “Oral Surgery Procedures” OR “Dental Surgery” OR “Maxillofacial Surgery” OR “Oral Surgical Procedures” OR “Oral and Dental Surgery” OR “Dental Oral Surgery” OR “Oral Surgical Interventions” OR “Oral Surgery Treatments” OR “Dental Maxillofacial Surgery” OR “Maxillofacial Oral Surgery” OR “Dental Surgical Procedures” OR “Oral and Facial Surgery” OR “Oral and Dental Surgical Interventions” OR “Oral and Maxillofacial Surgical Techniques” OR “Dental Surgical Treatments” OR “Surgical Procedures in Oral Medicine” OR “Dental and Maxillofacial Surgery” OR “Oral and Facial Surgical Procedures” OR “Surgical Interventions in Oral Care” OR “Postoperative Socket Irrigation” OR “Post-Surgery Socket Rinse” OR “Post-Surgical Socket Cleaning” OR “After Surgery Socket Irrigation” OR “Reduces Risk” OR “Decreases Risk” OR “Lowers Risk” OR “Minimizes Risk” OR “Inflammatory Complications” OR “Inflammation-Related Complications” OR “Complications due to Inflammation” OR “Inflammatory Issues” OR “Surgical Removal of Third Molars” OR “Third Molar Extraction” OR “Wisdom Teeth Removal” OR “Third Molar Surgery”).

### 2.3. Analysis Software

The R Studio software version 4.3.2 (2023-10-31 ucrt) was utilized for this study, given its capabilities as a programming language for statistical analysis and scientometric graphics. Additionally, CiteSpace 6.2.R7 was employed for its ability to visualize and analyze trends and patterns in scientific literature.

### 2.4. Data Analysis

Data from the 227 studies were scrutinized to assess scientific output in this domain, identify literature patterns and trends, and evaluate collaborations among authors, institutions, and countries. The study also examined spatiotemporal dynamics, which involved tracking changes in scientific production in this field over time and across different locations.

## 3. Results

This study was conducted over a 32-year period, from 1991 to 2023. During this time, 227 academic papers from 134 different sources, including both journals and books, were reviewed. The literature in this field has experienced an annual growth rate of 10.44%. The papers have an average age of 9.3 years and have received an average of 15.5 citations each. In total, these documents refer to 7227 different papers. Regarding the content of the papers, 773 additional keywords and 649 keywords provided by the authors were identified. A total of 975 different authors contributed to these papers, with 6 of them writing single-authored papers. On average, each paper was co-authored by 4.57 people, and 20.7% of the papers had international collaboration. Regarding the typology of the papers, the majority were articles (216), followed by early access articles (2), conference proceedings articles (3), editorial material (1), and reviews (5) (Table 1).

**Figure 1:**
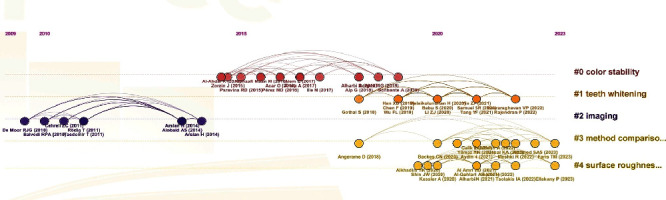
Timeline visualization.

The “Timeline Visualization” facilitated the identification of trends and patterns in the data. This analysis revealed four distinct clusters over time, suggesting the emergence of four main themes. Notably, the visualization highlighted a consistent increase in the number of publications and authors in 2010, accompanied by substantial collaborative activity. Key contributors during this period included De Moor (2010) and Balvedi (2010) (Figure 1). Furthermore, a renewed surge in interaction between publications and their citations was observed from 2020 onwards. Backes (2020) and Liz (2020) were among the most prominent authors in this later period (Figure 1).

**Figure 2:**
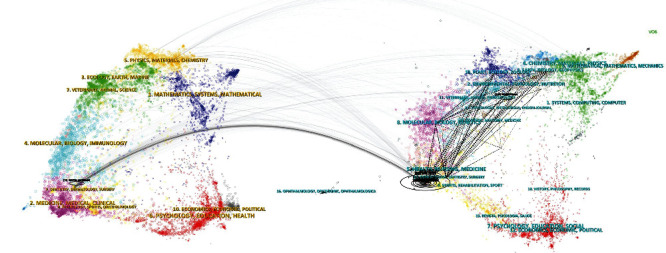
Dual map overlay. The continuous lines indicate the direction between the scientific disciplines and the disciplines in which publications are cited. Dashed lines indicate a large interaction between the citations of different fields or disciplines.

The “Dual Map Overlay” graph allowed for visualizing and analyzing the trends and patterns of scientific publications in this field. This graph showed two maps: one representing the disciplines of origin of the citations and the other the disciplines of destination. In this way, it can be observed how an origin influences the destination. The maps revealed a strong influence of the source disciplines (cluster 9 “Dentistry, Dermatology, Surgery”) on the target disciplines (clusters 5 “Health Nursing, Medicine” and 14 “Dentistry, Dermatology, Surgery”), indicating a significant flow of knowledge between these fields. In addition, a large, increasing trend in interdisciplinarity was observed, with an increasing number of citations with other clusters such as 2, 3, 10, and 11. This pattern suggests that the field of study is evolving towards an increasingly interdisciplinary nature (Figure 2).

**Figure 3:**
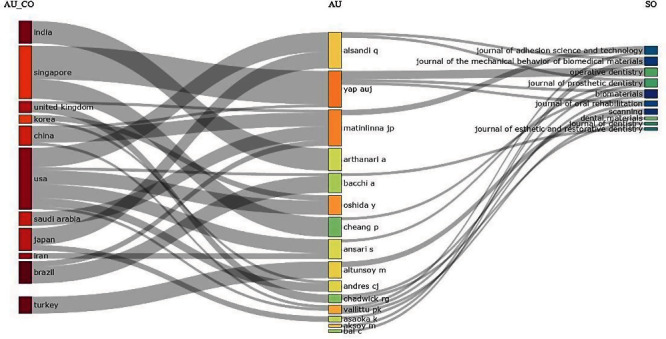
Three field plots.

The Sankey plot allowed us to analyze the relationship between three fields (country, author, and journal). This was useful to visualize the flow of information or the relationship between the different fields. Thus, a significant interaction was observed between the USA, Singapore, and India as the 3 most productive countries and the authors Yap, Aslandi, and Matinlinna. These findings provide a better understanding of the structure and evolution of this field (Figure 3).

**Figure 4:**
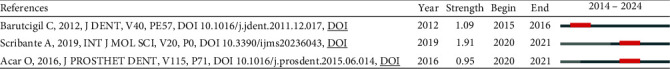
Top 3 references with the strongest citation burst.

The Citation Burst graph identified three references that have experienced the largest “burst” of citations in an evaluated time period. This evidenced a sudden and significant increase in the number of times these papers were cited. These studies correspond to Barutcigil (2012), Scribante (2019), and Acar (2016) (Figure 4).

**Figure 5:**
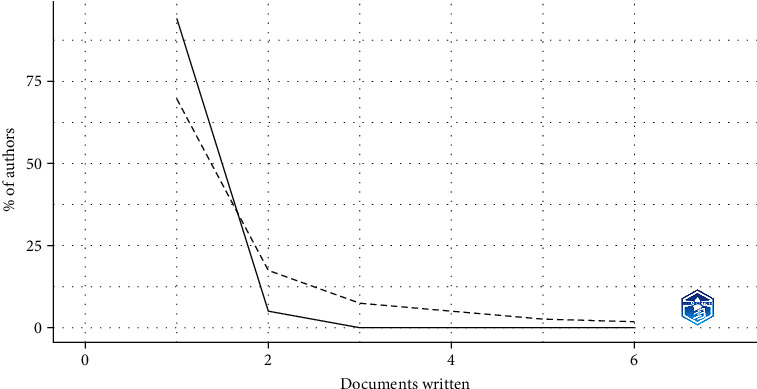
Author productivity (Lotka's law).

Lotka's law described the productivity of authors. It was found that most authors (922) have published a single paper, whereas a smaller number of authors (49) have published two papers, and only a few authors have published more than two papers. These findings suggest that, in this field of study, most authors contribute a small number of papers, while a small number of prolific authors contribute a large amount of the existing literature (Figure 5).

**Figure 6:**
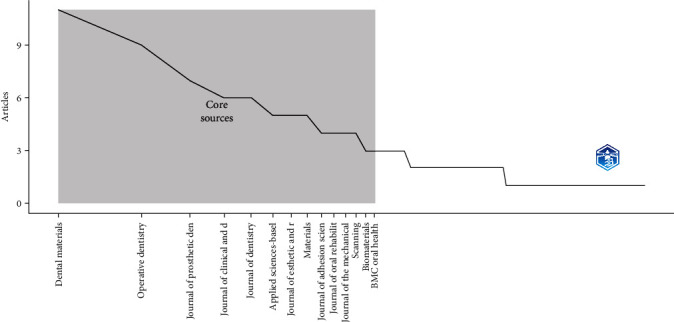
Core sources (Bradford's law).

Bradford's law showed that Zone 1 includes the most productive journals, and it was found that 13 journals have published a total of 73 articles. Zone 2, on the other hand, included 37 journals that have published a total of 70 articles. These findings suggest that a small number of journals contribute a large proportion of the literature in our field of study (Figure 6).

## 4. Discussion

In a dental office, tap water undergoes multiple treatment steps before use. Initially, it is filtered and stored in a reservoir, then decalcified using a high-performance resin [13]. The dental unit is a system that distributes water to various locations through a hydraulic system: patient cups, bottled water reservoirs, ultrasonic scalers, handpieces for high-speed drills, and air and water syringes [14]. All this is done to prevent infection and contamination. Water systems in residential and older buildings may be contaminated with *Legionella*, posing a potential risk to patients [15].

In addition, water is also important for overall oral health. Drinking sufficient water daily helps to maintain good oral health by preventing several oral conditions [16, 17]. Drinking water, which is a combination of desalinated water and groundwater, is provided at a reduced cost to most households [18]. Community water fluoridation has been recognized for its substantial contribution to enhancing dental health by reducing the prevalence of caries [19]. This approach is not only safe but also economically efficient in mitigating caries across large populations. However, water can serve as a carrier for bacteria, and an elevated concentration of fluoride in drinking water could result in dental fluorosis [20].

Despite progress in delivering safe drinking water, billions of people worldwide still lack access to this vital resource. The implications of this deficiency are alarming, as they correlate directly with preventable diseases and fatalities [21]. In this context, securing access to safe and sustainable drinking water has become a critical objective and an urgent challenge for global public health. Tackling this issue will not only enhance individual health and well-being but is also a fundamental requirement for human survival and prosperity. The provision of safe drinking water has surfaced as one of the most pressing public health challenges of our time [22, 23].

In recent years, technology has broadened its global influence. Reverse osmosis, a process that filters water through a dense membrane to segregate contaminants, removes all suspended particles, including organic residues, colloids, bacterial and viral contaminants, and ionic salts [24]. Consequently, reverse osmosis systems have been widely employed to enhance drinking water quality and ensure water supply, even in emergencies. In dentistry, the use of filtered water is paramount. It not only guarantees the safety and effectiveness of dental procedures but also aids in preventing oral and systemic diseases. As research and technology progress, it is crucial to continue exploring and optimizing water filtration methods to further enhance the quality of dental care [25].

Unlike our study, the bibliometric study on advanced oxidation processes (AOPs) by Macías-Quiroga et al. revealed research trends worldwide and in Ibero-America in the field of wastewater treatment. The 18,751 records were retrieved from Scopus and Web of Science. The information was meticulously sorted and sifted through, pinpointing authors and corresponding institutions. The study provided a visual representation of research trends, encompassing aspects such as the triennial publication count, country-wise publication distribution, continental participation, leading journals and authors, and the most cited institutions. It also offered a global co-author network and a visualization of the keyword network, underscoring the significant contribution of Ibero-America to worldwide research [26].

In contrast, a bibliometric study conducted by Ballesteros et al. on the topic of water disinfection using solar energy unveiled that innovative and sustainable alternatives such as solar water disinfection (SODIS) and solar photocatalysis are being employed. The USA, Spain, and China are leading in terms of publication output in this burgeoning field, while the most acclaimed research groups are based in Europe. The majority of the publications are centered around SODIS and photocatalytic nanomaterials. However, a smaller portion is dedicated to ensuring sufficient levels of water disinfection, testing regulated microbial indicators and emerging pathogens, and applying these methods in real-world scenarios [27].

Lastly, paralleling our study, a bibliometric analysis conducted by Chen et al. offered an extensive overview of research on wastewater treatment and emerging pollutants spanning from 1998 to 2021. The study amassed a total of 10,605 publications, with China leading in terms of publication count and exhibiting the closest collaboration with the United States. The most frequently cited papers underscored that purification or elimination techniques like ozonation or membrane filtration are effective in removing pharmaceutical compounds from water bodies [28].

Some potential limitations were presented in the present study. First, the findings are based only on the existing literature in WOS and, therefore, may be subject to potential selection bias. Second, international collaboration was measured only in terms of co-authorship, which may not necessarily fully reflect the nature and extent of collaboration at the global level. Third, although emerging trends and patterns were identified, these should be interpreted with caution, as the field of study is constantly evolving and trends may change over time. However, the present study had some strengths; for example, it provides a comprehensive overview of the evolution of scientific production in the use of filtered water in dentistry, covering a broad period of time. A detailed analysis of publications was performed, including collaboration between authors and countries, and emerging trends and patterns were identified, despite the constant evolution of the field. The use of advanced scientometric tools to analyze the data increases the accuracy and validity of the results. In addition, the study highlights the importance of international collaboration in this field, despite limitations in its measurement, which may foster future collaborations and advances in research.

The choice to extract data only from the Web of Science for this study is based on several reasons. Firstly, the Web of Science is one of the most comprehensive and reliable databases for scientific literature, spanning various disciplines and providing detailed citation information, which is crucial for a scientometric study. Secondly, it has a friendly user interface and offers advanced tools for data analysis, making it easy to extract and analyze data. However, we recognize that the exclusive use of the Web of Science may limit the coverage of the study, and we suggest that future research could consider the inclusion of other databases to obtain a more complete view.

## 5. Conclusion

Within the limitations of this study, it is concluded, first, that a detailed vision of the scientific production in the field of water filtration in dentistry is provided over a period of 32 years, which allows us to understand how this has evolved in the field over time. Second, by identifying emerging trends and patterns in the literature, this study can help researchers identify areas of interest and opportunities for future research. Furthermore, by highlighting international collaboration and knowledge sharing between different disciplines, this study underlines the increasingly interdisciplinary nature of this field. Lastly, by emphasizing the importance of water filtration in dentistry, this study may contribute to increasing awareness of the need to ensure the quality of water used in dental practices, which may have direct implications for patient safety and the effectiveness of the treatment. In summary, this review has the potential to inform and guide both clinical practice and future research in the field of dentistry. In addition, the study underscores the critical role of water filtration in patient safety and treatment efficacy, emphasizing the need for stringent quality control measures in dental practices. In essence, this review serves as a roadmap for advancing water filtration practices in dentistry, ensuring optimal patient care and treatment outcomes.

## Figures and Tables

**Table 1:** Main data information.

Description	Results
Main information about the data	
Timespan	1991–2023
Sources (journals, books, etc.)	134
Documents	227
Annual growth rate (%)	10.44
Document average age	9.3
Average citations per document	15.5
References	7227
Document contents	
Keywords plus (ID)	773
Author's keywords (DE)	649
Authors	
Authors	975
Authors of single-authored docs	6
Authors collaboration	
Co-authors per doc	4.57
International co-authorships (%)	20.7
Document types	
Article	216
Article: early access	2
Article: proceedings paper	3
Editorial material	1
Review	5

## Data Availability

The data are available upon request to the corresponding author.
